# Exploring the Safety and Efficacy of Glutathione Supplementation for Skin Lightening: A Narrative Review

**DOI:** 10.7759/cureus.78045

**Published:** 2025-01-27

**Authors:** Taif F Alzahrani, Shaykhah M Alotaibi, Albatool A Alzahrani, Atyaf F Alzahrani, Leen E Alturki, Muath M Alshammari, Raghad A Alharbi, Shuruq I Alanazi, Waleed Z Alshammari, Abdullah S Algarni

**Affiliations:** 1 Department of Dermatology, College of Medicine, University of Jeddah, Jeddah, SAU; 2 College of Pharmacy, King Saud University, Riyadh, SAU; 3 Department of Dermatology, College of Medicine, Majmaah University, Almajmaah, SAU; 4 Department of Dermatology, College of Medicine, Hail University, Hail, SAU; 5 Department of Dermatology, College of Medicine, Almaarefa University, Riyadh, SAU

**Keywords:** glutathione, hyperpigmentation, melanin reduction, skin lightening, skin whitening

## Abstract

The rising demand for skin-lightening products has brought glutathione, a tripeptide with antioxidant properties and melanogenesis-regulating effects, into focus as a potentially safer alternative to conventional agents. This narrative review aims to evaluate the efficacy and safety of oral, topical, and intravenous glutathione in skin-lightening therapies. Oral administration shows significant but variable decreases in melanin levels with limited side effects. Topical formulations provide good-level melanin reduction and skin texture improvement but with varying sustainability. Intravenous glutathione, although having rapid action, is associated with serious safety concerns like anaphylaxis and hepatotoxicity, further aggravated by a lack of standardized dosing protocols. Current evidence supports glutathione's potential as a depigmenting agent but underscores the need for rigorous, large-scale clinical trials to establish long-term safety, optimal dosing, and standardized applications. Until such data are available, clinicians and consumers should exercise caution to ensure safe and effective dermatological practices, particularly with intravenous use.

## Introduction and background

Fair skin is a prevalent aesthetic and cultural preference, particularly in many regions of Asia and Africa. Products designed to achieve this are widely used, primarily aimed at minimizing hyperpigmentation, reducing dark spots, and achieving uniform skin tone. However, the use of these products poses significant risks to human health, placing individuals in a vulnerable position. Notably, they are associated with an increased likelihood of developing serious health conditions, including a heightened risk of skin cancer, along with other adverse effects, such as premature aging, immune system suppression, and potential allergic reactions or chemical toxicity, depending on the specific product and its ingredients [1].

In response to these concerns, the use of glutathione has gained popularity as a safer alternative to harmful skin-lightening products. Glutathione is a tripeptide molecule composed of the amino acids cysteine, glycine, and glutamine. It plays a vital biological role as an essential component of the body’s defense system, combating free radicals and oxidative stress [2].

Glutathione has several applications in clinical therapeutics, particularly in managing conditions involving oxidative stress, such as neurodegenerative disorders, liver diseases, and inflammatory conditions, due to its ability to scavenge free radicals and reduce oxidative damage [3]. Melanogenesis is another process mediated by glutathione involving melanin production, i.e., the pigment responsible for skin color. The principle behind using glutathione in skin lightening lies in its ability to decrease melanin production, thereby lightening the skin and reducing hyperpigmentation. Additionally, studies have found lower glutathione levels in patients with sun-exposed lesions, such as actinic keratosis, and skin cancers, including basal cell carcinomas (BCCs). This reduction in glutathione levels can be attributed to its role as an antioxidant, as it is consumed in combating the oxidative stress induced by sun exposure [3].

The market for skin-lightening products is vast, with an economy of billions of dollars per annum globally. Among the various methods explored for skin health, oral and topical supplementation with glutathione have gained attention due to their proven efficacy and favorable safety profile compared to other approaches [4]. However, numerous studies on glutathione’s role in skin lightening lack comprehensive evaluations of long-term safety and efficacy. This highlights a significant gap in the literature and underscores the need for systematic reviews to clarify the precise role of glutathione in skin lightening [5].

Rationale of the study

The use of glutathione for skin lightening has been fueled by anecdotal evidence and early research that is promising in all aspects. Oral glutathione has been advertised as a safe and effective procedure for attaining fairer skin within a short time. However, despite all the progress, there exist specific gaps in understanding the facts and workings of glutathione levels for the induction of skin lightening. For example, glutathione's role in skin lightening is clearly understood. However, the primary mechanism underlying the maintenance of melanin levels in the skin and the change in skin tone is still unclear and has not been wholly explored [6].

Glutathione plays a critical role in melanogenesis by modulating several biochemical mechanisms, including reducing the oxidation level of tyrosinase, a key enzyme involved in melanin production. This regulatory function highlights glutathione's potential impact on pigmentation processes [7]. Glutathione is thought to influence the prevention of dark pigmentation spots by modulating melanin production; however, several population-specific factors can impact its effectiveness such as dosage, route of administration, consistency, and reproducibility [7].

In addition, some studies have highlighted the potent skin-lightening effects associated with glutathione utilization. However, the use of glutathione has also been scrutinized by multiple researchers due to concerns over its inconsistent outcomes and questions regarding its overall effectiveness [8]. The safety of both oral and topical glutathione supplementation remains a critical consideration, particularly with prolonged use. While it is generally regarded as safe in clinical practice, the potential systemic effects of long-term use have not yet been thoroughly researched or studied. Concerns include the possibility of toxicity and interactions with other medications. However, more serious or long-term side effects have not been comprehensively investigated to date [9]. Given the use of glutathione in cosmetic formulation and the enhanced demand for skin-lightening solutions, a detailed literature review is required to evaluate both the benefits and the harms of glutathione supplementation [10].

Aims/objectives

This narrative review aims to provide a comprehensive analysis of the safety and efficacy of glutathione supplementation for skin lightening. The specific objectives of this review are as follows.

Assess the Safety Profile of Glutathione Supplementation

Evidence surrounding the safety of glutathione supplementation-both short-term and long-term use is examined. Adverse effects, contraindications, and risks related to oral or intravenous glutathione supplementation for skin lightening will also be scrutinized. The review promises to cover cases documented with toxicity or side effects in clinical and non-clinical settings.

Effectiveness of Glutathione With Reference to Skin Lightening

An essential objective of this review is to critically evaluate existing studies about the outcome of topical or systemic application of glutathione for skin lightening. The studies will include measurements of lightning intensity, reduction of hyperpigmentation, and other types of skin color improvement. Comparative studies against other skin-lightening chemicals will also be considered to assess the relative effectiveness of glutathione as a skin-lightener.

Mechanisms by Which Glutathione Influences Melanin Production

Understanding how glutathione influences melanogenesis is paramount to defining its use for skin lightening. This review will also discuss the biochemical and molecular pathways that mediate glutathione interactions with the crucial enzymes that synthesize melanin, especially tyrosinase, alongside how its antioxidant properties will influence pigmentation modulation.

Identify Gaps in Contemporary Research and Suggest Future Directions

This review identifies gaps and elaborates on future directions.

## Review

Materials and methods

Study Selection

This narrative review employed a two-stage selection process to ensure the inclusion of relevant and high-quality studies. In the first stage, titles and abstracts of articles retrieved from databases such as PubMed and Google Scholar were screened. The selection criteria focused on the type of study (e.g., animal or human), dermatological conditions like hyperpigmentation, and safety considerations, including adverse effects.

The second stage thoroughly reviewed full-text articles identified during the initial screening. This stage assessed each study's eligibility based on predefined inclusion and exclusion criteria. The review included a detailed synthesis of study design, sample size, intervention methods (oral, intravenous, or topical glutathione), and key findings related to efficacy and safety. Studies were critically appraised, and their findings were synthesized narratively to provide a comprehensive understanding of glutathione's role in skin lightening.

Inclusion Criteria

The review included clinical trials, observational studies, and reviews investigating the use of glutathione for skin lightening. Priority was given to randomized controlled trials (RCTs), cohort studies, case-control studies, and systematic reviews. Review articles summarizing evidence from multiple studies on glutathione were also incorporated to offer a broader perspective on its efficacy and safety.

Studies were restricted to human subjects utilizing glutathione for body skin lightening, irrespective of the route of administration (oral, intravenous, or topical). Eligible studies specifically examined glutathione's effects on improving skin tone, reducing dark spots, treating hyperpigmentation, or enhancing overall skin appearance.

The inclusion criteria prioritized studies assessing the efficacy of glutathione supplementation in achieving measurable skin-lightening outcomes. Safety-related aspects, such as adverse effects, side effects, and toxicity, were also evaluated. Additionally, studies reporting changes in melanin production were considered essential for understanding the mechanism of action of glutathione on skin pigmentation.

Only studies published in English were included to ensure consistency and reliability in data synthesis. The review was restricted to publications from 2010 onwards to capture the most recent advancements in the clinical applications of glutathione.

Exclusion Criteria

Studies focusing exclusively on glutathione's antioxidant properties without relevance to skin lightening were excluded. Similarly, studies investigating glutathione's effects on general health or unrelated conditions, such as liver health or immune function, were not considered. 

Research not utilizing glutathione as an intervention was excluded, as this review specifically targeted its skin-lightening application. Studies failing to report key outcomes, such as efficacy, safety, adverse effects, or toxicity, were also excluded to maintain the focus on relevant findings.

Animal studies and in vitro research were excluded, as this review concentrated solely on human subjects. Duplicate publications presenting the same data were identified and excluded to avoid redundancy. Lastly, studies published in languages other than English were excluded, as their relevance could not be assessed within the scope of this review.

Results

Efficacy of Glutathione Supplementation for Skin Lightening

Oral glutathione: Oral glutathione supplements have demonstrated mixed results in clinical studies, prompting further research to explore the effects of varying dosages, treatment durations, and individual factors [11]. A randomized, double-blind, placebo-controlled trial reported a significant reduction in melanin indices after four weeks of supplementation with 500 mg/day, particularly in sun-exposed areas [12]. However, Allen and Bradley reported no significant changes in melanin indices or oxidative stress biomarkers, emphasizing the variability in observed outcomes [13]. Scientists have investigated the potential of oral glutathione for overall skin lightening. In a randomized, double-blind, placebo-controlled trial, Arjinpathana and Asawanonda demonstrated that participants taking 500 mg of oral glutathione daily exhibited significantly lower melanin levels in sun-exposed areas, such as the face and wrists, compared to the control group [12]. These findings suggest that oral glutathione may be effective in lightening various skin types.

Wahab et al. demonstrated that the effectiveness of oral glutathione is enhanced when combined with other topical agents, the combined oral and topical use of topical glutathione produced more significant skin-lightening effects as compared to the use of a single agent [14]. Similarly, Duperray et al. found that 2400 mg of oral glutathione with 300 mg L-cystine resulted in significant improvement in lightening of facial skin, as well as significantly reduced dark spots [15]. This finding again supports the hypothesis that combination therapies will yield more synergistic mechanisms to help with the skin-lightening effect, but more studies are warranted to validate the synergistic effects of glutathione further [15].

Variability in outcomes across studies can be attributed to a range of factors, including demographic, genetic, and environmental differences, as highlighted by various studies [16,17]. Additionally, the dosage and duration of supplementation play critical roles in determining the extent of skin-lightening effects. Oral glutathione is generally well-tolerated, with reported side effects including transient gastrointestinal discomfort that typically resolves on its own [7,16]. Overall, oral glutathione shows promise as a systemic skin-lightening agent, particularly when optimized for dosage and duration or used alongside complementary therapies. However, more studies are needed to find the best ways to use it and to see if it has long-term effects.

Topical glutathione: Topical glutathione has demonstrated effectiveness in reducing hyperpigmentation, particularly in conditions such as melasma. In a double-blind, randomized, placebo-controlled study, Watanabe et al. observed that the twice-daily application of 2% glutathione lotion over 10 weeks significantly reduced melanin indices compared to the placebo group. Additionally, the study reported notable improvements in skin moisture and texture, highlighting the multifunctional benefits of topical glutathione [18].

Patients with melasma exhibited marked improvement with topical glutathione. A 67.4% reduction in the mMASI score after 90 days of treatment with 2% glutathione was reported [19]. Improvements were also observed in the Physician Global Assessment (PGA) and quality of life scores, supported by photographic evidence of reduced pigmentation and enhanced skin tone [19].

Etnawati et al. further confirmed the efficacy of glutathione in skin brightening, noting that skincare products containing 0.5% glutathione were more effective than those with 0.1%, achieving significant improvements in skin brightness and hyperpigmented spots within eight weeks [20]. In another study, Wahab et al. demonstrated that combining topical and oral glutathione provided superior results compared to either method alone [21].

Microneedling has been shown to enhance the effects of topical glutathione. Mohamed et al. found that combining microneedling with topical glutathione accelerated and improved skin lightening compared to microneedling alone [22]. Similarly, Sarkar et al. reported that 2% topical glutathione used alongside microneedling outperformed lower concentrations and controls in reducing melasma spots [23].

The mechanisms underlying these effects include the inhibition of tyrosinase activity, reduction of reactive oxygen species (ROS), and modulation of melanin production, shifting from eumelanin to lighter pheomelanin [8]. These findings highlight the potential of topical glutathione as a safe and effective treatment for hyperpigmentation, mainly when combined with complementary therapies such as microneedling or oral supplementation.

Intravenous Glutathione: Poor efficacy and considerable safety concerns make intravenous (IV) glutathione one of the most controversial methods for skin lightening. Zubair et al. reported that 37.5% of participants receiving 1200 mg IV glutathione twice a week for six weeks claimed they had lighter skin compared to 18.7% in the placebo group [24]. However, these effects were short-lived, with benefits fading after six months. Additionally, 32% of participants experienced adverse events, including liver dysfunction and one case of anaphylaxis, raising significant doubts about the safety of this method [24].

The Philippine Food and Drug Administration (FDA) has issued warnings against IV glutathione, citing risks such as liver damage, severe allergic reactions, and the absence of standardized dosing protocols [25]. While IV administration results in higher plasma concentrations than oral or topical methods, the lack of regulatory oversight and comprehensive safety data limits its clinical use. A study by Sarkar et al. further cautioned against IV glutathione, emphasizing the insufficient evidence supporting its efficacy and the heightened safety risks [26].

In summary, while IV glutathione may offer short-term skin-lightening effects, these benefits are outweighed by its risks and transient outcomes. More studies are needed to develop standardized protocols, evaluate long-term safety, and examine their effectiveness in diverse populations.

Esterified Derivatives of Glutathione

Esterified derivatives of glutathione, such as glutathione monoethyl ester (GSH-MEE), offer advantages over traditional oral glutathione (GSH), which is poorly absorbed due to degradation in the digestive tract [27]. These derivatives demonstrate improved cell penetration and stability, enabling more effective suppression of tyrosinase activity and a subsequent reduction in melanin production. Additionally, esterification addresses the limitations associated with conventional glutathione, potentially leading to more consistent and reliable therapeutic outcomes [27]. Additionally, Pouchieu et al. reported that after 12 weeks of treatment, esterified glutathione combined with polyphenol-rich formulations significantly reduced tyrosinase activity and improved the appearance of hyperpigmented spots [28]. This combination also improves the action of glutathione derivatives in tackling pigmentation problems. However, safety remains a concern. Cytotoxicity has been demonstrated in preclinical studies for derivatives such as glutathione diethyl ester (GSH-DEE) and glutathione mono-isopropyl ester (GSH-MIPE), which could limit their clinical applicability [27]. Although preclinical data suggest promising effects, most of them were performed in vitro or on small sample sizes, which requires additional studies to assess their safety and efficacy in more extensive clinical settings. In conclusion, esterified derivatives of glutathione show greater potential as effective chemical agents for skin lightening as compared to free glutathione. These derivatives can overcome the significant bioavailability challenges that limit the efficacy of traditional glutathione, potentially leading to more efficient and potent treatments. However, larger, well-designed clinical studies are essential to validate their long-term safety and optimize their application in dermatologic practice.

Safety of Glutathione Supplementation

Oral and topical glutathione: Oral and topical glutathione are generally well-tolerated, with mild and transient side effects reported in some studies. Gastrointestinal symptoms, such as flatulence and loose stools, were the most common side effects of oral glutathione, as observed by Weschawalit et al. and Sharma et al. [3,22]. These symptoms were not severe and typically resolved without intervention during treatment periods ranging from 4 to 10 weeks. Similarly, topical glutathione demonstrated a favorable safety profile. In a study by Watanabe et al., a 2% glutathione lotion caused no adverse events [6].

Research by Wahab et al. and Etnawati et al. further corroborated the safety of oral and topical glutathione, with no serious side effects noted in their respective trials [14,21]. Minor complaints, such as mild tingling or abdominal discomfort, were infrequent and transient [21]. Petit and Rahiman et al. highlighted the importance of educating consumers to address psychosomatic perceptions of side effects, particularly those using glutathione for cosmetic purposes [24,25].

A study by Methaq and Mehmood emphasized the rare nature of side effects associated with oral and topical formulations [16]. Direct application of topical glutathione appears particularly safe, with its effects confined to the treated areas [16]. Furthermore, glutathione's antioxidant properties enhance skin elasticity and provide protection against UV-induced damage, as highlighted by Boo et al. [29]. Studies by Sarkar et al. and Sitohang et al. have also affirmed the overall safety of oral and topical glutathione when used in accordance with recommended protocols [7,17]. However, Naidoo et al. raised concerns about misusing unregulated products in regions with weak consumer protections, underscoring the need for proper manufacturing and safe usage guidelines [26]. In conclusion, oral and topical glutathione is safe for cosmetic and therapeutic use when properly formulated and applied, with rare and mild adverse effects.

Intravenous glutathione: Intravenous glutathione has been a topic of considerable debate due to its associated safety concerns. Unlike oral and topical formulations, which are generally well-tolerated, the injectable form poses significant risks [30]. Studies have reported serious adverse effects, including liver failure in almost a third of the patients, allergic reactions, and one case of anaphylaxis, prompting discontinuation of the treatment in some cases [18,19]. Consequently, these data indicated the potential hazard of indiscriminate usage, especially in cosmetic applications, citing serious risks, including hepatic injury and hypersensitivity, and that glutathione should not be sold outside the hospital setting [30].

Furthermore, systematic reviews showed that there are no established superior effects of systemic delivery over topical or oral routes for skin-lightening, raising questions on the overall benefit-risk profile of this route [28]. In light of these risks and the lack of robust clinical evidence, the administration of intravenous glutathione should be restricted to controlled environments with proper regulatory controls. Its safety, effectiveness, and dosing protocols should be thoroughly investigated before recommending it for broader use.

Discussion

Glutathione is pivotal in pigmentation regulation, making it a valuable agent in skin-lightening therapies [2]. It acts primarily by inhibiting tyrosinase, a key enzyme in melanin synthesis, by binding to copper at its active sites, thereby reducing the production of eumelanin, the darker pigment [31]. Furthermore, glutathione promotes the conversion of eumelanin to pheomelanin, a lighter yellow-red pigment, by facilitating cysteine conjugation with dopaquinone [31]. This dual mechanism contributes to overall skin lightening and addresses hyperpigmentation associated with excessive eumelanin production [31].

In addition to these mechanisms, glutathione modulates melanocyte activity by downregulating the microphthalmia-associated transcription factor (MITF), which controls tyrosinase expression and melanocyte proliferation [29]. This regulation balances melanin production, reducing unwanted pigmentation [29]. Compared to other lightening agents, such as hydroquinone or kojic acid, which primarily inhibit tyrosinase activity [32], glutathione offers additional benefits through its antioxidant and anti-inflammatory properties, including the inhibition of interleukin-6 (IL-6) and prostaglandins that drive inflammation-induced melanogenesis [31]. These unique mechanisms make it particularly useful in managing post-inflammatory hyperpigmentation, where inflammation plays a central role.

Oral Glutathione: Efficacy and Safety

Oral glutathione has demonstrated promising results in skin-lightening therapies, with multiple studies confirming its efficacy in reducing melanin indices. Arjinpathana and Asawanonda found significant reductions in melanin levels after four weeks of supplementation at 500 mg/day [12]. Similarly, Handog et al. reported that 90% of participants experienced moderate skin-lightening effects after eight weeks of 500 mg/day glutathione lozenges [20].

Despite these successes, some studies, like Allen and Bradley, observed no significant changes in melanin indices, highlighting outcome variability [13]. Individual differences, dosage, and treatment duration likely contribute to this inconsistency. While oral glutathione is generally well-tolerated, mild gastrointestinal side effects, including flatulence and loose stools, have been reported but resolve spontaneously [7].

Oral glutathione remains a promising systemic skin-lightening agent, especially when combined with other interventions. Future research should standardize dosing and assess its long-term safety profile to optimize its clinical application.

Topical Glutathione: Efficacy and Safety

Topical glutathione has proven effective in reducing hyperpigmentation, particularly in conditions like melasma [18]. Watanabe et al. reported significant reductions in melanin indices after 10 weeks of twice-daily application of 2% glutathione lotion [6]. Supporting evidence from the literature showed a 67.4% reduction in the Modified Melasma Area Severity Index (mMASI) score, along with improvements in the PGA and Melasma Quality of Life (MELASQOL) scores [19]. Photographic documentation further validated these findings, revealing visibly reduced hyperpigmentation and enhanced skin tone [19].

Combination therapies enhance the efficacy of topical glutathione. Mohamed et al. found that microneedling with glutathione yielded superior results compared to microneedling alone [23]. Similarly, Etnawati et al. noted more significant improvements in skin brightness with higher concentrations of glutathione in topical formulations [21]. Mechanistically, topical glutathione inhibits tyrosinase activity and reduces oxidative stress, shifting melanin production toward pheomelanin [29].

Topical glutathione is well-tolerated, with only mild localized reactions, such as erythema, reported in a small percentage of participants [28]. These findings underscore its safety and efficacy, especially when combined with systemic or procedural therapies.

Intravenous Glutathione: Efficacy and Safety

Intravenous (IV) glutathione is controversial due to significant safety concerns despite its potential for rapid skin-lightening effects. Zubair et al. reported temporary skin-lightening effects in 37.5% of participants receiving 1200 mg IV glutathione twice weekly for six weeks; however, 32% experienced adverse events, including liver dysfunction and one case of anaphylaxis [18]. The absence of standardized dosing protocols and potential misuse further exacerbate these concerns. Given the significant risks and lack of robust efficacy data, IV glutathione should be restricted to carefully monitored clinical settings [33]. Stricter regulatory oversight and further research are essential to ensure safety and establish standardized protocols.

Photographic Evidence and Tolerability

Photographic evidence from studies such as those by Watanabe et al. visually confirmed the efficacy of glutathione in reducing hyperpigmentation and improving skin tone [6]. These findings were supported by high tolerability across studies, with only mild, transient adverse events reported for oral and topical formulations [29]. The absence of severe adverse drug reactions reinforces the safety profile of these routes of administration.

## Conclusions

The biological mechanisms of glutathione, combined with consumer demand for safer alternatives to conventional skin-lightening agents, have contributed to its increasing popularity for skin-lightening purposes. Various studies have demonstrated significant potential, particularly with oral and topical formulations. Oral supplementation has shown measurable, though sometimes inconsistent, skin-lightening effects and a favorable safety profile at standard dose levels. Topical formulations have been associated with significant improvements in hyperpigmentation and skin quality, with minimal and self-resolving side effects. In contrast, intravenous glutathione raises serious safety concerns, including the risk of anaphylaxis and hepatotoxicity, compounded by the lack of standardized protocols and rigorous clinical studies. These factors highlight that, while glutathione can be an effective skin-lightening agent, especially in its oral or topical forms. Additional large, well-designed clinical trials are needed to address critical gaps. These include long-term safety, optimal dosing, and the durability of its skin-lightening effects.

Until such data become available, caution is advised regarding the use of glutathione, particularly in its intravenous form, which should be restricted to closely supervised clinical settings. The unregulated use of glutathione presents potential risks, emphasizing the need for robust regulatory oversight. Ultimately, the integration of glutathione into dermatological practice should be guided by a balance between its therapeutic potential and safety considerations, supported by evidence-based clinical guidelines.
